# Supplementary material for: Hidden spin-orbital texture at the Γ¯-located valence band maximum of a transition metal dichalcogenide semiconductor
Source: Nat Commun. 2022 Jul 16;13:4147. doi: 10.1038/s41467-022-31539-2 (PMC9288546; doi:10.1038/s41467-022-31539-2)
Supplement: Supplementary file 1 — Supplementary Information [file 41467_2022_31539_MOESM1_ESM.pdf]

Supplementary Information for:
Hidden spin-orbital texture at the $\overline{\Gamma}$-located valence band maximum of a transition metal dichalcogenide semiconductor

Oliver J. Clark,[1,†] Oliver Dowinton,[2] Mohammad Saeed Bahramy,[2] and Jaime Sánchez-Barriga[1,3]

[1]*Helmholtz-Zentrum Berlin für Materialien und Energie, Elektronenspeicherring BESSY II, Albert-Einstein-Str. 15, 12489 Berlin, Germany*
[2]*Department of Physics and Astronomy, University of Manchester, Oxford Road, Manchester M13 9PY, United Kingdom*
[3]*IMDEA Nanoscience, C/ Faraday 9, Campus de Cantoblanco, 28049, Madrid, Spain*

Note 1: Disparity in $p_{x,y}$ and p_z bandwidths along k_z

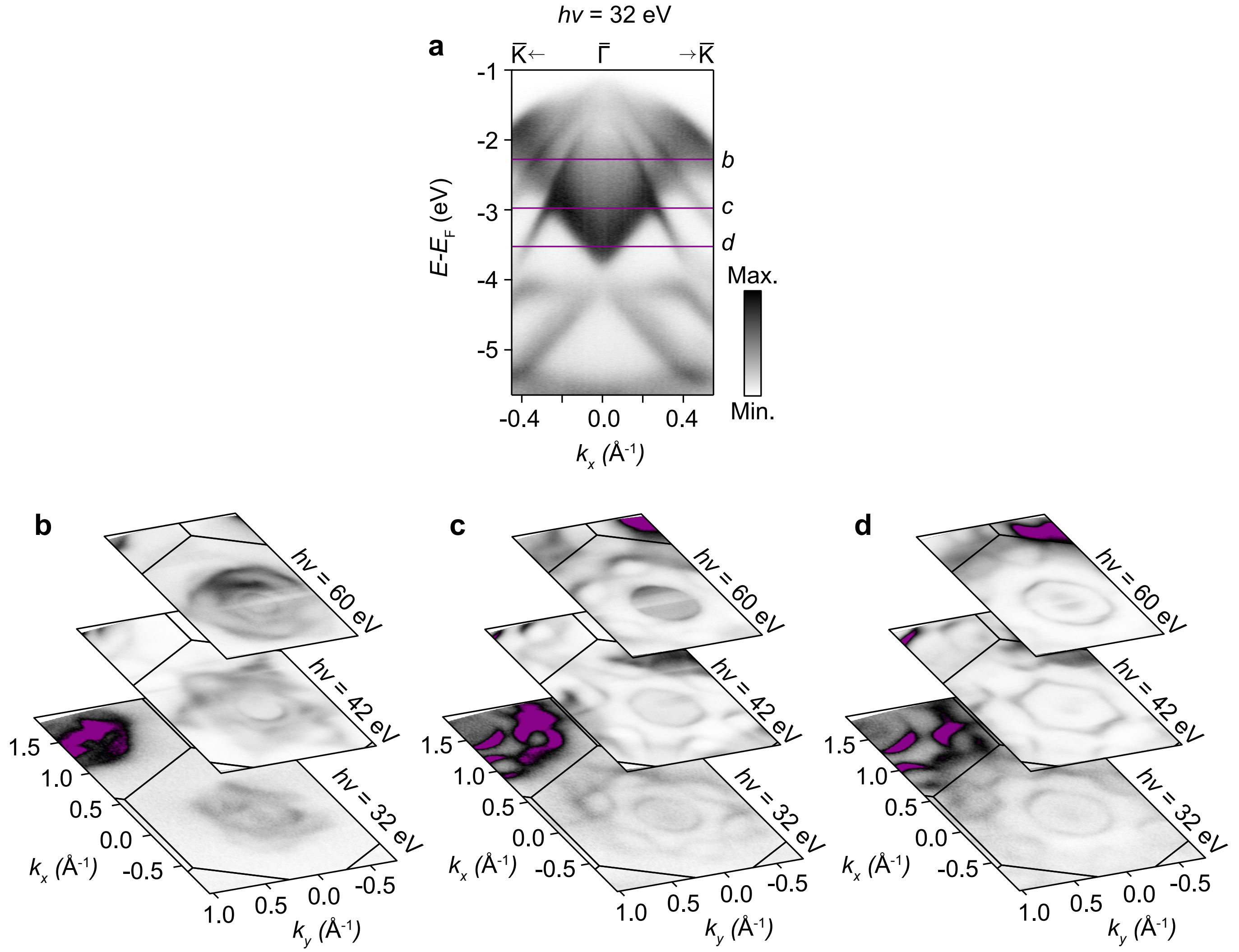

FIG. 1. **Three-dimensionality of $p_{x,y}$-derived states** (a) Dispersion probing close to the Γ plane along the $\overline{\mathrm{K}}$ - $\overline{\Gamma}$-$\overline{\mathrm{K}}$ direction. Horizontal lines indicate the energies for the constant-energy k_x,k_y,k_z contours shown in (b-d). (b-d) Constant energy contours for the energies indicated in (a). The photon energies used to probe each layer are indicated, with 32, 42 and 60 eV photons probing close to Γ, A and Γ planes respectively. Note the periodicity in the shape of the $p_{x,y}$-derived bands around the $\overline{\Gamma}$ point as a function of k_z (photon energy).

Note 2: Tight-binding model for the determination of orbital contributions to local spin polarisation

The existence of time-reversal (TR) symmetry, implies invariance of our energy eigen-states under an anti-unitary operator of the form

$$\mathcal{T} = \mathcal{U}_T \Theta, \tag{1}$$

where $\mathcal{U}_T$ is a unitary operator and Θ is complex conjugation. For spinors we require $\mathcal{T}^2 = -\mathcal{I}$, hence we choose $\mathcal{U}_T = i\sigma_y$ on the spinor sub-space, without loss of generality. This requires the Bloch wave functions to satisfy $T\psi_{\uparrow\downarrow}(k) = \psi^*_{\downarrow\uparrow}(-k)$ At $k = 0$, *i.e.* the Γ point, leading to a global spin degeneracy $\varepsilon_\uparrow = \varepsilon_\downarrow$, regardless of whether the system is subject to any further symmetry constraints or not. However, this constraint is not locally valid. As such, each atomic centre may hold a net spin polarisation as long as a counterpart can cancel this contribution, thus keeping the whole system non-magnetic. Realising this situation requires some sort of directional anisotropy in the crystal field. In $HfSe_2$, this condition is fulfilled by the trigonal C_{3v} symmetry of the lattice. The resulting crystal field splits each Se-p group into two sub-manifolds $\{p_x, p_y\}$ and p_z, which, when mixed by the spin-orbit interaction, form three doubly degenerate $|J, \pm m_j\rangle$ branches with $J = 3/2$ and 1/2, and $1/2 \leq m_j \leq J$.

We can use these $|J, m_j\rangle$ states to form a 12-band tight-binding model to describe the Se-p orbital-dominated valence bands of $HfSe_2$, $\psi_v = \sum_{J,m_j} \alpha_{J,m_J} |J, m_j\rangle$. The explicit form of the resulting basis set arising from one of the Se atoms is listed in Table I (adopted from Ref. [1, 2]).

TABLE I. The $|J, m_j\rangle$ basis set chosen for each Se ion in $HfSe_2$. Below $x, y,$ and z corresponds to p_x, p_y and p_z orbitals respectively and the two spinors are indicated by arrows.

$\lvert J, m_j\rangle$	Spin-orbital expression
$\left\lvert \frac{3}{2}, +\frac{3}{2} \right\rangle$	$\frac{1}{\sqrt{2}} \left\lvert (x+iy)\uparrow \right\rangle$
$\left\lvert \frac{3}{2}, +\frac{1}{2} \right\rangle$	$\frac{1}{\sqrt{6}} \left\lvert (x+iy)\downarrow \right\rangle - \sqrt{\frac{2}{3}} \left\lvert z\uparrow \right\rangle$
$\left\lvert \frac{3}{2}, -\frac{1}{2} \right\rangle$	$-\frac{1}{\sqrt{6}} \left\lvert (x-iy)\uparrow \right\rangle - \sqrt{\frac{2}{3}} \left\lvert z\downarrow \right\rangle$
$\left\lvert \frac{3}{2}, -\frac{3}{2} \right\rangle$	$\frac{1}{\sqrt{2}} \left\lvert (x-iy)\downarrow \right\rangle$
$\left\lvert \frac{1}{2}, +\frac{1}{2} \right\rangle$	$\frac{1}{\sqrt{3}} \left\lvert (x+iy)\downarrow \right\rangle + \sqrt{\frac{1}{3}} \left\lvert z\uparrow \right\rangle$
$\left\lvert \frac{1}{2}, -\frac{1}{2} \right\rangle$	$-\frac{1}{\sqrt{3}} \left\lvert (x-iy)\uparrow \right\rangle + \sqrt{\frac{1}{3}} \left\lvert z\downarrow \right\rangle$

With this model, we can now easily compute all three components $i = \{x, y, z\}$ of spin polarisation localised at each Se site, a, using $S_{i,a} = \langle \psi_{v,a} | \sigma_i | \psi_{v,a} \rangle$, where σ_i are Pauli spin matrices. As an example, let us find $S_{y,\mathrm{Se1}}$ at the Γ point. For the spin degree of freedom, α, the time reversal (TR) operator $\mathcal{T}_\alpha$ obeys the properties:

$$\begin{aligned} \text{TR} : |\uparrow\rangle &\longrightarrow \mathcal{T}_\alpha |\uparrow\rangle = -|\downarrow\rangle \\ \text{TR} : |\downarrow\rangle &\longrightarrow \mathcal{T}_\alpha |\downarrow\rangle = |\uparrow\rangle \\ \text{TR} : \sigma_y &\longrightarrow \mathcal{T}_\alpha \sigma_y \mathcal{T}_\alpha{}^{-1} = -\sigma_y. \end{aligned} \tag{2}$$

Here the sign of the spin under TR is in a sense arbitrary, however the switch in sign is significant. For the orbital degrees of freedom, β we have:

$$\begin{aligned} \text{TR} : |x+iy\rangle &\longrightarrow \mathcal{T}_\beta |x+iy\rangle = -|x-iy\rangle \\ \text{TR} : |x-iy\rangle &\longrightarrow \mathcal{T}_\beta |x-iy\rangle = -|x+iy\rangle \\ \text{TR} : |z\rangle &\longrightarrow \mathcal{T}_\beta |z\rangle = |z\rangle \end{aligned} \tag{3}$$

allowing us to define a natural total TR operator $\mathcal{T}_{\alpha,\beta}$ over our J basis states. Consider the matrix element M_1

$$M_1 = \langle x-iy, \uparrow | x+iy, \uparrow \rangle = \int (Y_1^{-1})^* Y_1^1. \tag{4}$$

Where Y_l^m are spherical harmonics. From TR symmetry we can see

$$M_1 = \langle x+iy, \downarrow | x-iy, \downarrow \rangle = \int (Y_1^1)^* Y_1^{-1} = \left(\int (Y_1^{-1})^* Y_1^1 \right)^*, \tag{5}$$

which implies that M_1 is real. Now suppose that the σ_y matrix element

$$\langle x-iy,\uparrow|\sigma_y|x+iy,\downarrow\rangle = -i\int (Y_1^{-1})^* Y_1^1 = -iM_1 \tag{6}$$

is non-vanishing. Under TR this goes to

$$\langle x+iy,\downarrow|\sigma_y|x-iy,\uparrow\rangle = i\int (Y_1^1)^* Y_1^{-1} = i\left(\int (Y_1^{-1})^* Y_1^1\right)^* = iM_1^*, \tag{7}$$

which implies M_1 is imaginary, so by contradiction this σ_y matrix element must be zero. For the σ_y matrix element

$$\langle x+iy,\uparrow|\sigma_y|x+iy,\downarrow\rangle = -i\int (Y_1^1)^* Y_1^1 = -iM_2, \tag{8}$$

M_2 is clearly real, but again under TR the σ_y matrix element goes

$$\langle x-iy,\downarrow|\sigma_y|x-iy,\uparrow\rangle = i\int (Y_1^{-1})^* Y_1^{-1} = i\int (Y_1^1)^* Y_1^1 = iM_2, \tag{9}$$

where the last step follows from the fact $(Y_1^{-1})^* = -Y_1^1$, implying M_2 is imaginary, such that this σ_y element must be zero by contradiction. Hence the only non-zero σ_y elements are those of form

$$\langle x \pm iy,\uparrow\downarrow|\sigma_y|z,\downarrow\uparrow\rangle. \tag{10}$$

As such, we find that there are only four overlap integrals with finite values as follows:

$$\begin{aligned} S_y^1 &= 2A\left\langle \frac{3}{2},+\frac{3}{2}\middle|\sigma_y\middle|\frac{3}{2},-\frac{1}{2}\right\rangle = 2A\left\langle \frac{3}{2},+\frac{1}{2}\middle|\sigma_y\middle|\frac{3}{2},-\frac{3}{2}\right\rangle = +\frac{4iA}{\sqrt{3}}\left\langle x+iy\middle|z\right\rangle \\ S_y^2 &= 2B\left\langle \frac{3}{2},+\frac{3}{2}\middle|\sigma_y\middle|\frac{1}{2},-\frac{1}{2}\right\rangle = 2B\left\langle \frac{1}{2},+\frac{1}{2}\middle|\sigma_y\middle|\frac{3}{2},-\frac{3}{2}\right\rangle = -\frac{4iB}{\sqrt{6}}\left\langle x+iy\middle|z\right\rangle \end{aligned} \tag{11}$$

where $A = \alpha^*_{|3/2,+3/2\rangle}\alpha_{|3/2,-1/2\rangle}$ and $B = \alpha^*_{|3/2,3/2\rangle}\alpha_{|1/2,-1/2\rangle}$. Of course, the spatial integral $\langle x+iy|z\rangle$ in Eq. 11, if taken over the whole real space, amounts to zero, but if limited to a small region around Se1, can still remain finite. Furthermore, given that $A \neq B$, the local $S_{y,\mathrm{Se1}} = S_y^1 + S_y^2$ is also non-zero. The contributions from S_y^1, S_y^2 and $S_y^1 + S_y^2$ are shown in Fig. 5 of the main text. Within the same framework, we can similarly show $S_{y,\mathrm{Se2}} = -S_{y,\mathrm{Se1}}$, enforcing the TR symmetry on the whole system. A close inspection of Eq. 11 reveals that the orbital mixing of the in-plane $p_{x\uparrow\downarrow}$ and $p_{y\uparrow\downarrow}$ orbitals with the out-of-plane $p_{z\downarrow\uparrow}$ orbital is the driving force behind this hidden spin polarisation. It also signifies the critical importance of directional anisotropy in the crystal field and its delicate but profound interplay with spin-orbit coupling.

Note 3: Probing the time-reversal symmetric spin texture of p-orbital derived bands with p-polarised light

Consider a p-derived band, or collection of p-derived bands, at the $\overline{\Gamma}$ point of the surface Brillouin zone. Let the band carry a spin-polarisation with vector $\vec{S} = \vec{S}_{p_x} + \vec{S}_{p_y} + \vec{S}_{p_z}$, where $\vec{S}_{p_i}$ are the p-orbital resolved ($i = \{x, y, z\}$) contributions to the overall spin vector $\vec{S}$.

We can write the individual components of $\vec{S} = S_x\hat{x} + S_y\hat{y} + S_z\hat{z}$ as

$$S_x = \sum_{i=\{x,y,z\}} S_{p_i,x}\cos^2(\theta) + \sum_{i=\{x,y,z\}} S_{p_i,y}\sin^2(\theta) = 0 \tag{12}$$

$$S_y = \sum_{i=\{x,y,z\}} S_{p_i,x}\sin^2(\theta) + \sum_{i=\{x,y,z\}} S_{p_i,y}\cos^2(\theta) = 0 \tag{13}$$

$$S_z = \sum_{i=\{x,y,z\}} S_{p_i,z} = 0 \tag{14}$$

The azimuthal angle θ is a sample rotation in the x-y plane.

Eqns. (1-3) carry two constraints in order to comply with time-reversal symmetry. Firstly, spin vectors measured at $(k,\theta) = (+k, 0)$ and $(-k, \pi)$ are equivalent in the presence of time-reversal symmetry. For $k = 0$, a π sample rotation should then leave any measured spin polarisation unchanged. Secondly, each component of $\vec{S}$ must be equal to zero at the time-reversal invariant momentum point, $\overline{\Gamma}$, with each of the sums $\sum_i S_{p_i,x}$, $\sum_i S_{p_i,y}$ and $\sum_i S_{p_i,z}$ individually amounting to zero.

Now consider the selection rules imposed by the the linearly polarised light. The photoemission matrix element is dependent only on the parity of the orbitals with respect to the scattering plane, and is therefore dependent on the azimuthal angle θ of the sample. For $p-$polarisation and $\theta = 0$, only p_x and p_z orbitals are probed. Following a rotation of $\theta = \pi/2$, p_y and p_z orbitals are probed.

We can write out the measured spin-components with $p-$polarised light for $\theta = 0$ and $\theta = \pi/2$.

$$S_x(\theta = 0) = S_{p_x,x} + S_{p_z,x} \tag{15}$$

$$S_y(\theta = 0) = S_{p_x,y} + S_{p_z,y} \tag{16}$$

$$S_z(\theta = 0) = S_{p_x,z} + S_{p_z,z} \tag{17}$$

$$S_x(\theta = \frac{\pi}{2}) = S_{p_y,y} + S_{p_z,y} \tag{18}$$

$$S_y(\theta = \frac{\pi}{2}) = S_{p_y,x} + S_{p_z,x} \tag{19}$$

$$S_z(\theta = \frac{\pi}{2}) = S_{p_y,z} + S_{p_z,z} \tag{20}$$

Summing Eqn. 4 and Eqn. 8 and making use of $\sum_i S_{p_i,x} = 0$:

$$S_x(0) + S_y(\frac{\pi}{2}) = S_{p_x,x} + S_{p_z,x} + S_{p_y,x} + S_{p_z,x} = S_{p_z,x} \tag{21}$$

Summing Eqn. 6 and Eqn. 9 and making use of $\sum_i S_{p_i,z} = 0$:

$$S_z(0) + S_z(\frac{\pi}{2}) = S_{p_x,z} + S_{p_z,z} + S_{p_y,z} + S_{p_z,z} = S_{p_z,z} \tag{22}$$

In the case where the band is entirely $p_{x,y}$ derived, or there is no p_z contribution to the spin vector ($S_{p_z,x} = S_{p_z,y} = S_{p_z,z} = 0$), the sums in Eqns. 10 and 11 evaluate to zero. The measured spin-polarisations in the x and y channels then obey the relation $S_x(\theta) = -S_y(\theta + \frac{\pi}{2})$. In addition, the measured spin polarisation in the $S_z(\theta)$ channel becomes symmetric about $S_z = 0$ ($S_{p_y,z} = -S_{p_x,z}$) with period π.

In the case where there is a p_z contribution, $S_z(\theta)$ will remain periodic but with a constant offset corresponding to the value of $S_{p_z,z}$ which is measured equivalently at all θ. The behaviour of $S_x(\theta)$ and $S_y(\theta)$ is more complex, with the summation $S_x(\theta) + S_y(\theta + \pi/2)$ periodic between limits determined from $S_{p_z,x}$ and $S_{p_z,y}$.

We note that all spin functions should possess π/n periodicity (where n is an integer) in order to yield equivalent observations for $\theta = 0$ and $\theta = \pi$, where the orbital-dependent matrix elements are equivalent. This condition does not hold for a time-reversal symmetry breaking spin texture.

An analogous description can be made for s-polarised photons, resulting in opposite orientations for the measured spin-polarisation in each of the x, y and z channels in the absence of a p_z contribution.

† Corresponding author. E-mail address: oliver.clark@helmholtz-berlin.de

[1] G. Bastard, *Wave mechanics applied to semiconductor heterostructures* (Les Editions de Physoque, Le Ulis, 1998).
[2] L. C. Voon, and M. Willatzen, *The k.p Method*, (Springer, Berlin 2009).
